# Dosimetric effect of multileaf collimator leaf width in intensity-modulated radiotherapy delivery techniques for small- and large-volume targets

**DOI:** 10.4103/0971-6203.79690

**Published:** 2011

**Authors:** S. A. Yoganathan, Karthick Raj Mani, K. J. Maria Das, Arpita Agarwal, Shaleen Kumar

**Affiliations:** Department of Radiotherapy, Sanjay Gandhi Postgraduate Institute of Medical Sciences, Lucknow, India

**Keywords:** Dynamic, intensity-modulated radiotherapy, multileaf collimator, step-and-shoot IMRT

## Abstract

The purpose of this study was to evaluate the dosimetric effect of the leaf width of a multileaf collimator (MLC) in intensity-modulated radiotherapy (IMRT) delivery techniques for small- and large-volume targets. We retrospectively selected previously treated 5 intracranial and 5 head-neck patients for this study to represent small- (range, 18.37-72.75 cc; mean, 42.99 cc) and large-volume (range, 312.31-472.84 cc; mean, 361.14 cc) targets. A 6-MV photon beam data was configured for Brianlab m3 (3 mm), Varian Millennium 120 (5 mm) and Millennium 80 (10 mm) MLCs in the Eclipse treatment-planning system. Sliding window and step-shoot IMRT plans were generated for intracranial patients using all the above-mentioned MLCs; but due to the field size limitation of Brainlab MLC, we used only 5-mm and 10-mm MLCs in the head-and-neck patients. Target conformity, dose to the critical organs and dose to normal tissues were recorded and evaluated. Although the 3-mm MLC resulted in better target conformity (mean difference of 7.7% over 5-mm MLC and 12.7% over 10-mm MLC) over other MLCs for small-volume targets, it increased the total monitor units of the plans. No appreciable differences in terms of target conformity, organ at risk and normal-tissue sparing were observed between the 5-mm and 10-mm MLCs for large-volume targets. The effect of MLC leaf width was not quantifiably different in sliding window and step and shoot techniques. In addition, we observed that there was no additional benefit to the sliding-window (SW) technique when compared to the step-shoot (SS) technique as a result of reduction of MLC leaf width.

## Introduction

The development of inverse planning systems and methods for delivering nonuniform radiation intensities have ushered in the era of intensity-modulated radiation therapy (IMRT), representing the state of the art in the treatment of many cancers.[1] IMRT is one of the conformal radiotherapy techniques, which modulates the beam to create a conformal dose distribution around the target while minimizing dose to the surrounding normal tissues and enables tumor dose escalation. There are two ways of modulating the beam intensity in traditional IMRT — sliding-window (SW) / dynamic IMRT (continuous beam modulation) and step-shoot (SS) / multiple static segment (sequential exposure of sub-beams or segments) IMRT[2].

Multileaf collimator (MLC) is an important tool for beam modulation in IMRT delivery, which is available with different leaf widths. Since its introduction, the MLC leaf width has been reduced (e.g., from 10 mm to 2.5 mm) with the expectation to yield improvement in target conformity and normal-tissue sparing. The geometric changes in the MLC imply dosimetric considerations in IMRT, because the fluence map corresponding to a modulated field depends on how the leaves can shape the area of interest while protecting the normal tissue as well.[3] Abundant literatures are available on the dosimetric effect of the leaf width of MLC in IMRT on different sites.[3–10] These literatures reveal that significant improvement in plan quality was observed by using narrower leaves. These studies are limited to a particular IMRT delivery technique (either in SW or SS delivery). As the two IMRT techniques are different by their nature of delivery, the effect of MLC leaf width may also differ. There is no head-to-head comparison of the effect of MLC leaf width between SW and SS IMRT techniques.

In this study, we attempted to evaluate the difference in dosimetric impact of MLC leaf width between SW and SS IMRT techniques in small- and large-volume targets.

## Material and Methods

### Configuration of multileaf collimator models

A 6-MV photon beam data was configured in the Eclipse treatment planning system (Varian Medical Systems, Palo Alto, CA) with Brianlab m3 (3 mm), Varian Millennium 120 (5 mm) and Millennium 80 (10 mm) MLCs. The physical characteristics of these MLCs are tabulated in Table 1. The smallest leaf width (3 mm) used in this study is Brianlab m3 MLC (Brianlab AG, Munich, Germany). The medium (5 mm) and the widest (10 mm) leaf width MLCs used were Millennium 120 and Millennium 80 (Varian Medical Systems, Palo Alto, CA), respectively. To study only the dosimetric effect of leaf width, we intentionally used the same MLC dosimetric parameters (average leaf transmission, 2%; and static leaf gap, 2 mm) for all the configured MLC models.

**Table 1:** Physical characteristics of different multileaf collimators

*MLC model*	*Number of leafs*	*Maximum field size*	*Leaf width*	*Leaf speed (cm/s)*
Brainlab m3 (3 mm)	52	10 × 10 cm^2^	3 mm, 4.5 mm and 5.5 mm	1.5
Millennium 120 (5 mm)	120	40 × 40 cm^2^	Center 20-cm field: 5 mm	2.5
			Outer 20-cm field: 10 cm	
Millennium 80 (10mm)	80	40 × 40 cm^2^	10 mm	2.5

The above table shows the physical characteristics of the 3-mm, 5-mm and 10-mm MLCs used in this study

### Patient group

#### Small-volume group

We retrospectively selected 5 previously treated intracranial (Pituitary Adenoma) patients to replicate the small-volume (SV) targets with the mean target volume of 43 cc (range, 18.37-72.75 cc). The T1-weighted magnetic resonance images (2 mm slice thickness) were fused with contrast-enhanced radiotherapy planning CT images (2 mm slice thickness) to aid the delineation of the gross tumor volume (GTV). A symmetric margin of 5 mm was given to GTV to generate planning target volume (PTV). The prescription dose was 45 Gy in 25 fractions.

#### Large-volume group

We retrospectively selected 5 previously treated head-neck (stage T1T2, N0) patients for this study to replicate large-volume (LV) targets. GTV, Clinical Target Volume CTV and PTV were delineated in the contrast-enhanced CT images (3 mm slice thickness). All patients were planned for simultaneous integrated boost (SIB) utilizing a single plan to irradiate gross and microscopic disease at different dose-per-fraction values. The high-risk PTV (HR PTV) included the gross disease, and the low-risk PTV (LR PTV) was defined as the uninvolved adjacent lymph nodes at risk.[11,12] The mean target volume (both HR + LR PTV) was 362 cc (range, 298-443 cc). The prescription dose was 70 Gy in 35 fractions for HR PTV and 56 Gy in 35 fractions for LR PTV. Both the parotid glands and spinal cord were considered as critical organs.

### Treatment planning

The Eclipse treatment planning system, version 8.1 (Varian Medical Systems, Palo Alto, CA), was used in this study. Each of the SV group patients was planned for IMRT using all the three configured MLCs. To ensure fair comparison, all plans of a particular patient were optimized individually with the same dose constraint parameters. As the prescription dose of SV target is below the tolerance dose of the i.e. the optic chiasm, and the location of the organis within the PTV, no OAR was considered during optimization. The dose volume optimizer (DVO) inverse planning algorithm (Ver. 8.1) was used to obtain the optimal fluence. The optimal fluences were converted into actual fluences by leaf motion calculator (LMC) for both SW and SS delivery techniques. The beamlet size used by the treatment planning system is not user configurable (i.e., beamlet width in the direction of leaf motion is fixed at 2.5 mm, and perpendicular to the direction of leaf motion is the MLC leaf width).

The number of intensity modulation levels in SS technique specifies the resolution of converting an idealized intensity-modulated fluence distribution into a deliverable fluence map. Higher the number of modulation levels, greater is the number of monitor units and segments required for delivery.[13] It is recommended to have 10 or more intensity levels to produce dose distributions comparable with those in the SW technique. So all plans of SS technique were generated with 15 levels of intensity modulation.

Parameters such as the number of beams, gantry angles and field sizes were kept constant for a particular case. The dose calculation was performed using pencil beam convolution algorithm (Ver. 8.1) for a grid size of 2.5 mm with modified Batho’s heterogeneity correction.

In LV cases, due to the field size limitation (maximum, 10 × 10 cm^2^) of 3-mm MLCs, only 5-mm and 10-mm MLCs could be used to generate SW and SS IMRT plans. The parotid glands and spinal cord were considered as OARs in the optimization process. All plans were normalized at the target mean dose.

All plans were accepted with the criterion that at least 95% isodose envelope should encompass the entire PTV without any hot spot above 107%. The parotid glands were constrained to receive a mean dose of < 26Gy; whereas for spinal cord, the maximum dose was restricted to below 45 Gy.

The 95% dose volume, 50% dose volume, target volume, parotid glands mean dose, spinal cord maximum dose, and total monitor units were recorded for evaluation.

### Plan quality indices

The plan quality was evaluated in terms of target conformity, OAR and normal-tissue sparing. The following indices were used.

Conformity Index (CI)[14,15] was used to assess the target coverage.

$$\mathrm{Conformity}–\mathrm{Index}–\left(CI\right)–=–\frac{Ref–Isodose–Volume}{Target–Volume}$$

The 95% isodose volume was taken as reference isodose volume. The CI value is 1 for an ideal plan. In case of LV group patients, the CI was calculated separately for HR PTV and LR PTV.

The normal-tissue dose irradiation by different MLCs was evaluated by using the Normal-Tissue Dose Factor (NTDF).

$$\mathrm{Normal}-\mathrm{Tisssue}–\mathrm{Dose}–\mathrm{Factor}–\left(NTDF\right)–=–\frac{50\%–Isodose–Volume}{Target–Volume}$$

In order to compare the differences in dosimetric impact of MLC leaf width in SW and SS, the ratios of conformity indices, OAR doses and NTDFs between the two plans with different MLCs in the same IMRT technique were computed using the following formula.[7]

$$\mathrm{plan}–\mathrm{Quality}–\mathrm{Index}–\mathrm{Ratio}–\left(PQIR\right)–=–\frac{Quality–Index–of–one–MLC–plan}{Quality–Index–of–other–MLC–plan}$$

For example, the plan quality index ratios (PQIRs) of CI and NTDF for 3-mm and 5-mm MLC plans in SW IMRT were calculated as follows:

PQIR _CI 3 mm / 5 mm_= CI of 3-mm plan / CI of 5-mm plan.

PQIR _NTDF 3 mm / 5 mm_= NTDF of 3-mm plan / NTDF of 5-mm plan.

## Results

### Small-volume targets

**Figure 1:**
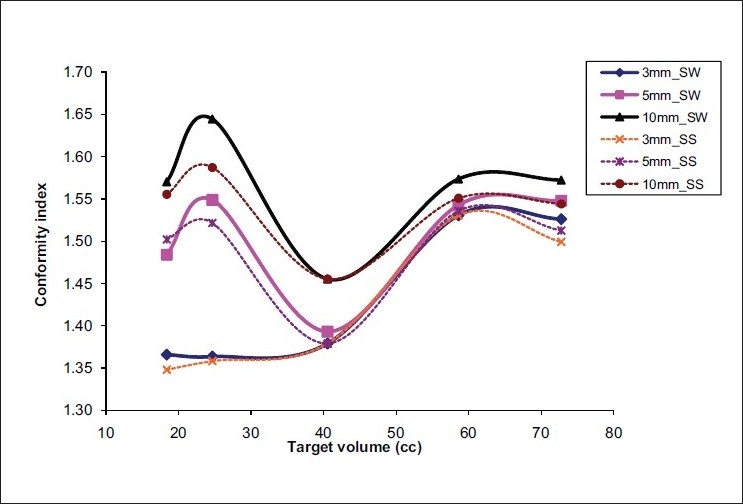
CI of SV targets for 3-mm, 5-mm and 10-mm MLCs in the two IMRT techniques

**Figure 2:**
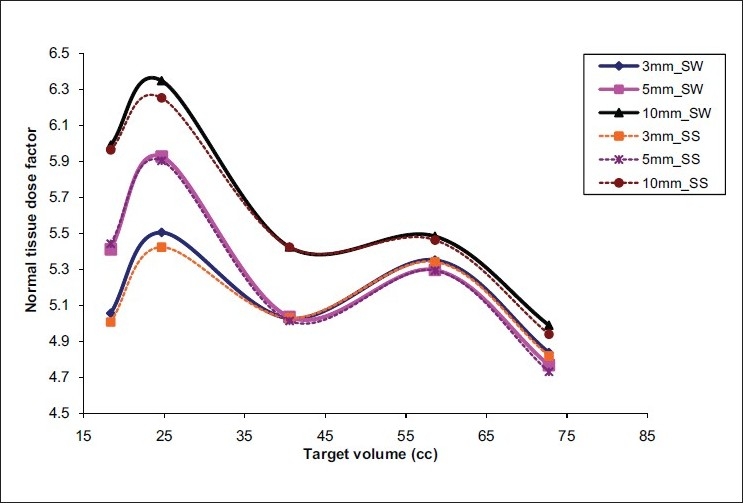
NTDF of SV targets for 3-mm, 5-mm and 10-mm MLCs in the two IMRT techniques

**Figure 3:**
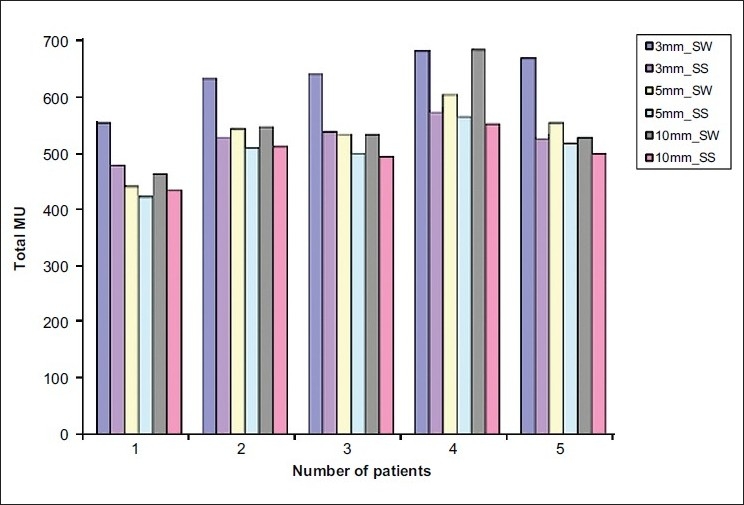
Total MUs of 3-mm, 5-mm and 10-mm MLCs in SV targets

The 3-mm MLC plans resulted in better target conformity and normal-tissue sparing when compared to 5-mm and 10-mm MLC plans in both the IMRT techniques. The average conformity index (mean ± SD) of 3-mm, 5-mm and 10-mm MLCs was 1.39 ± 0.08, 1.50 ± 0.08, and 1.57 ± 0.07 for SW IMRT; and 1.38 ± 0.08, 1.49 ±0.07, and 1.54 ± 0.05 for SS IMRT; respectively. The conformity index was superior for smaller leaf widths; but as the volume of the target increased, the difference in conformity indices of the MLCs decreased [Figure 1]. There was no appreciable difference between 3-mm and 5-mm leafs in terms of NTDF; but 10-mm leaf was found to irradiate a little more of normal tissue [Figure 2]. The ‘mean ± SD’ value of NTDF for 3-mm, 5-mm and 10-mm MLCs was 5.19 ± 0.281, 5.29 ± 0.44, and 5.65 ± 0.53 for SW IMRT; and 5.16 ± 0.30, 5.28 ± 0.44, and 5.61 ± 0.51 for SS IMRT; respectively. The 3-mm IMRT plans always required increased total number of monitor units (MUs) compared to other MLCs [Figure 3].

When comparing the SW and SS techniques, the 95% and 50% volumes were always less for SS technique. Consequently, the CI and NTDF values of SS technique were slightly better than those of SW technique. The PQIR values for various MLCs were calculated to compare the leaf width effects in SW and SS techniques [Table 2]. Overall, though the PQIR values were about the same in both the IMRT techniques, the magnitude of PQIR was slightly more in SW technique.

**Table 2:** Plan quality index ratio of the three multileaf collimators for small-volume targets

*Evaluation parameters*	*Mean PQIR of 10-mm and 3-mm MLC plans*		*Mean PQIR of 10-mm and 5-mm MLC plans*		*Mean PQIR of 5-mm and 3-mm MLC plans*	
	*SW*	*SS*	*SW*	*SS*	*SW*	*SS*
PTV CI	1.126	1.115	1.046	1.035	1.079	1.078
NTDF	1.088	1.087	1.068	1.063	1.021	1.019

The above table shows the plan quality index ratio (PQIR) of 3-mm, 5-mm and 10-mm MLC plans in SW and SS IMRT techniques. The PQIR values are slightly more for sliding-window technique

### Large-volume targets

The conformity index (mean ± SD) of 5-mm and 10-mm MLCs for HR PTV was 1.40 ± 0.17 and 1.45 ± 0.19 for SW IMRT; and 1.40 ± 0.17 and 1.44 ± 0.20 for SS IMRT; respectively. Similarly, for LR PTV, the value of CI of 5-mm and 10-mm MLCs was 1.64 ± 0.39 and 1.72 ± 0.41 for SW IMRT; and 1.67 ± 0.46 and 1.70 ± 0.44 for SS IMRT; respectively. These results revealed that the values of CI of 5-mm and 10-mm MLCs were not substantially different for large-volume targets.

Both the MLCs were able to provide the mean dose of parotid glands below 26 Gy. The average right parotid gland mean dose for 5-mm and 10-mm MLCs was 24.32 ± 2.30 Gy and 25.37 ± 2.42 Gy for SW IMRT; and 23.87 ± 2.05 Gy and 24.95 ± 2.08 Gy for SS IMRT; respectively. Whereas the average left parotid gland mean dose for 5-mm and 10-mm MLCs was 22.33 ± 1.90 Gy and 23.05 ± 2.10 Gy for SW IMRT; and 22.03 ± 1.71 Gy and 22.76 ± 1.85 Gy for SS IMRT; respectively.

Our results demonstrated that the dose to parotid glands and that to spinal cord were not quantifiably different. The NTDF values and total MUs were almost the same for both the MLCs.

**Figure 4:**
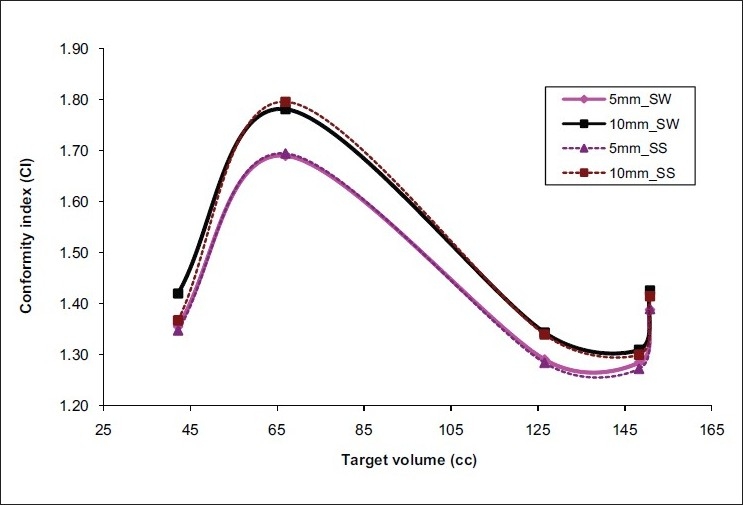
CI of LV targets (HR PTV) for 5-mm and 10-mm MLCs in the two IMRT techniques

Like SV targets, the 95% and 50% volumes were always less in SS technique for LV group patients; correspondingly the CI and NTDF values in SS technique were slightly better than those in SW technique. The PQIR values for 5-mm and 1-mm MLCs were calculated for the two IMRT techniques [Table 3]. The 5-mm and 10-mm MLCs had almost similar results with regard to target conformity [Figure 4], OAR and normal-tissue sparing in both the IMRT techniques.

**Table 3:** Plan quality index ratio of 5-mm and 10-mm multileaf collimators for large-volume targets

*Evaluation parameters*	*PQIR of 5-mm and 10-mm MLC plans*	
**	*SW*	*SS*
HR PTV CI	1.034	1.032
LR PTV CI	1.052	1.024
Left parotid gland mean dose	1.032	1.031
Right parotid gland mean dose	1.043	1.044
NTDF	1.037	1.034

The above table shows the plan quality index ratio (PQIR) of 5-mm and 10-mm MLC plans in SW and SS IMRT techniques. The PQIR values are little more for sliding-window technique

## Discussion

The 3-mm MLC could produce better target conformity (average difference of 7.7% over 5mm and 12.7% over 10mm MLC) and normal-tissue sparing in SV targets, and this advantage diminished as the target volume increased [Figure 1]. However, this relation may not be true for targets with irregular shapes and needs a separate study.

The 3-mm IMRT plans always required increased total number of monitor units [Figure 3]. As the beamlet size of optimizer is determined by the MLC width (perpendicular to the leaf motion direction), plans with smaller leaf widths have more number of beamlets. This leads to increment of total number of MUs in MLC plans with smaller leaf widths. Further, the lower leaf speed (maximum, 1.5 cm/s) of the 3-mm MLC was also a rationale for rise in total number of MUs, especially in SW delivery. In case of LV targets, the quality indices of 5-mm and 10-mm plans did not differ much, and they did not show any noticeable relation with target volumes.

In addition, the CI, OAR and NTDF values of SS technique were slightly better when compared to SW technique. This is attributed to fewer MUs required and the fact that change of field shapes in SS technique is only when the beam is kept off. Whereas in SW technique, the beam is continuously switched on, which increases dose to OAR and normal tissues due to transmission through leaves. Moreover, The SW delivery cannot completely shield any area; rather it sweeps the area with minimal gap and maximum leaf speed possible.[16,17]

As the SW and SS IMRT delivery techniques are different by their nature, one may intuitively think that the leaf width effect may also vary. Therefore, we compared the leaf width effect between SW and SS IMRT techniques as well. We calculated the ratio of plan indices between the MLC plans for comparison. We found that the effect of MLC leaf width was not demonstrably different between the two techniques and there was no additional benefit to the SW technique when compared to the SS technique as a result of reduction of MLC leaf width.

However, the accuracy of the results presented here may vary with parameters such as optimization algorithms, shape of the target (irregularity) and location of the OAR. The SS delivery technique will be more close to optimal fluence with increased intensity levels. Hence the intensity level of the SS technique may also modify the results.

## Conclusions

Dosimetric effects of MLC leaf width were studied for small- and large-volume targets in SW and SS IMRT techniques. The 3-mm MLC has the best conformity among all the MLCs in case of small-volume targets, but it increases the total number of MUs. The 5-mm and 10-mm MLCs resulted in similar plan-quality indices in case of large-volume targets. When comparing SS and SW techniques, the effect of the MLC leaf width is nearly the same in both the techniques.
